# Crystal structure and Hirshfeld surface analysis of 1-(furan-2-yl)-2-(thio­phene-2-carbon­yl)-3-(thio­phen-2-yl)-2,3,3a,8a-tetra­hydro­cyclo­penta­[*a*]inden-8(1*H*)-one

**DOI:** 10.1107/S2056989026005141

**Published:** 2026-05-19

**Authors:** Hayreddin Gezegen, Gamze Ordu, Zeliha Atioğlu, Mehmet Akkurt, Hamouda Adam Hamouda

**Affiliations:** a Department of Nutrition and Dietetics, Faculty of Health Sciences, Cumhuriyet University, 58140 Sivas, Türkiye; b Cumhuriyet University, Institute of Science, Department of Physics, 58140 Sivas, Türkiye; c Department of Aircraft Electrics and Electronics, School of Applied Sciences, Cappadocia University, Mustafapaşa, 50420 Ürgüp, Nevşehir, Türkiye; d Department of Physics, Faculty of Sciences, Erciyes University, 38039 Kayseri, Türkiye; e Department of Chemistry, Faculty of Science, University of Kordofan, Sudan; University of Neuchâtel, Switzerland

**Keywords:** crystal structure, chalcone derivatives, cyclo­penta­ne, thio­phene ring, furan ring

## Abstract

In the title compound, C_25_H_18_O_3_S_2_, the terminal cyclo­pentane ring adopts an envelope conformation. In the racemic crystal, pairs of enanti­omers are linked by C—H⋯O hydrogen inter­actions, forming *R*^2^_2_(10) ring motifs. The (1*R*,2S,3*S*,3a*R*,8a*S*) and (1*S*,2*R*,3*R*,3a*S*,8a*R*) enanti­omers are also connected by further C—H⋯O inter­actions, forming ribbons propagating along the *a*-axis direction. In addition, C—H⋯π inter­actions between these ribbons form layers parallel to the (001) plane. Crystal cohesion is ensured by van der Waals inter­actions between layers. The furan ring and the two thio­phene rings are disordered by a 180° rotation, with ratios of 0.728 (15):0.272 (15), 0.972 (3):0.028 (3) and 0.791 (3):0.209 (3), respectively. According to Hirshfeld surface analysis, the most important contributions for the crystal packing are H⋯H (52.8%), C⋯H/H⋯C (26.1%) and O⋯H/H⋯O (14.8%) inter­actions.

## Abstract 2

In the crystal, pairs of mol­ecules are connected by C—H⋯O hydrogen inter­actions, generating *R*^2^_2_(10) ring motifs. Pairs of mol­ecules are further linked by C—H⋯O inter­actions, forming ribbons propagating along the *a*-axis direction. C—H⋯π inter­actions between these ribbons create layers parallel to the (001) plane. van der Waals inter­actions between the layers maintain the cohesion of the crystal structure.

## 1. Chemical context

Chalcones and chalcone-like compounds (Karimi-Sales *et al.*, 2018 ▸; Singh *et al.*, 2014 ▸; Karaman *et al.*, 2010 ▸) possess high chemical activity in addition to their biological activity. The *α,β*-unsaturated carbonyl system present in their structure allows the synthesis of many new and polyfunctional compounds (Nair *et al.*, 2018 ▸; Ramya, *et al.*, 2018 ▸; Gezegen, 2017 ▸). Chalcone-type compounds are therefore very valuable compounds for organic chemists and can be used as a key component or valuable building block for achieving mol­ecular diversity. 2-Benzyl­idene-1-indanone derivatives are functional compounds containing an α,β-unsaturated carbonyl system. As a result of the presence of acidic methyl­ene protons in their structures, they undergo two consecutive Michael addition reactions in basic media. In our previous study, by reacting 2-benzyl­idene-1-indanone derivatives with chalcone derivatives in a basic medium, we synthesized a series of racemic deriv­atives featuring fused rings and five different stereocentres through Michael/Michael cascade addition reactions (Gezegen *et al.*, 2021 ▸). In this paper we report the crystal structure and Hirshfeld surface analysis of the title compound, C_25_H_18_O_3_S_2_, obtained in high yield from the reaction of a chalcone derivative with a chalcone-like compound.
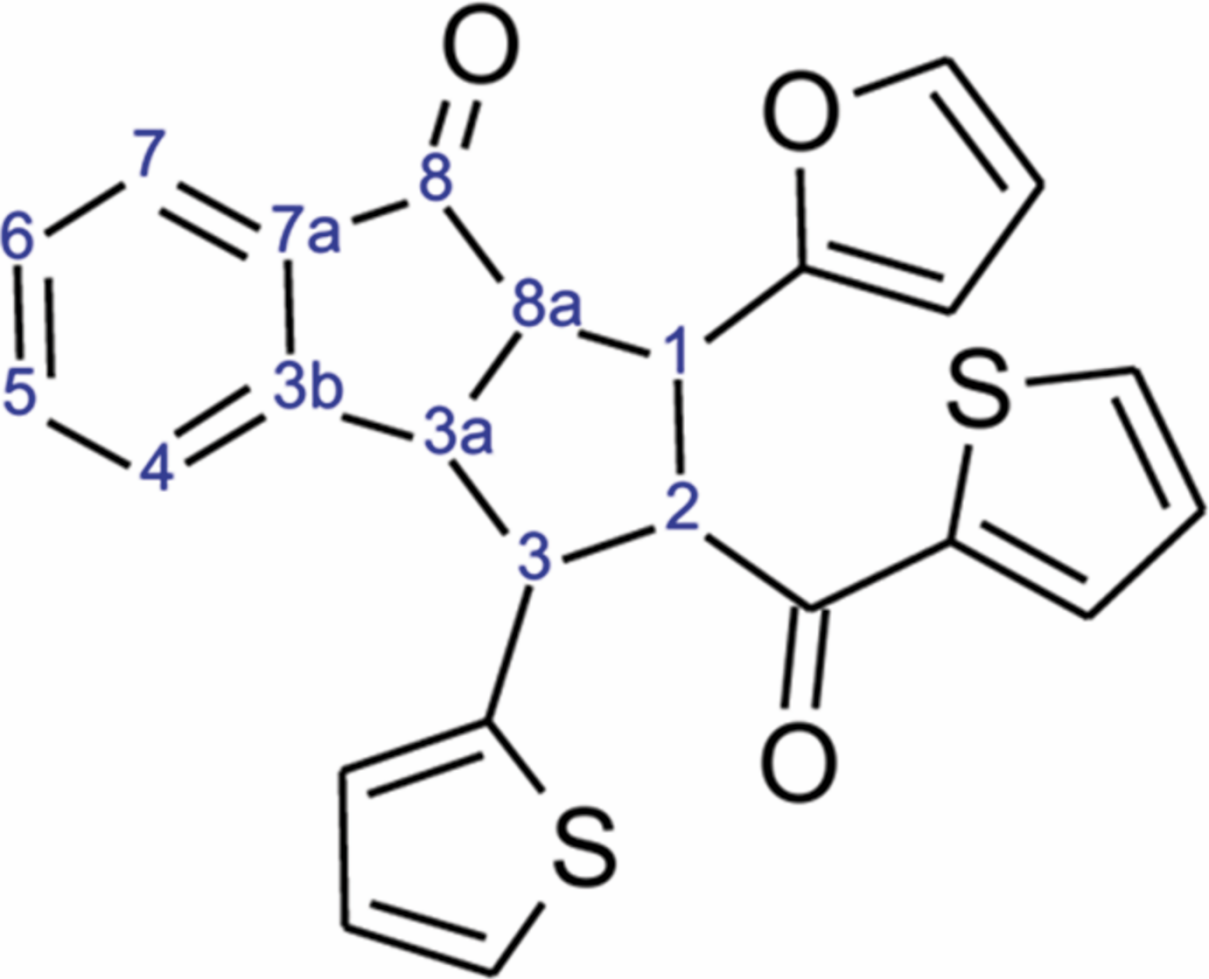


## 2. Structural commentary

**Figure 1:**
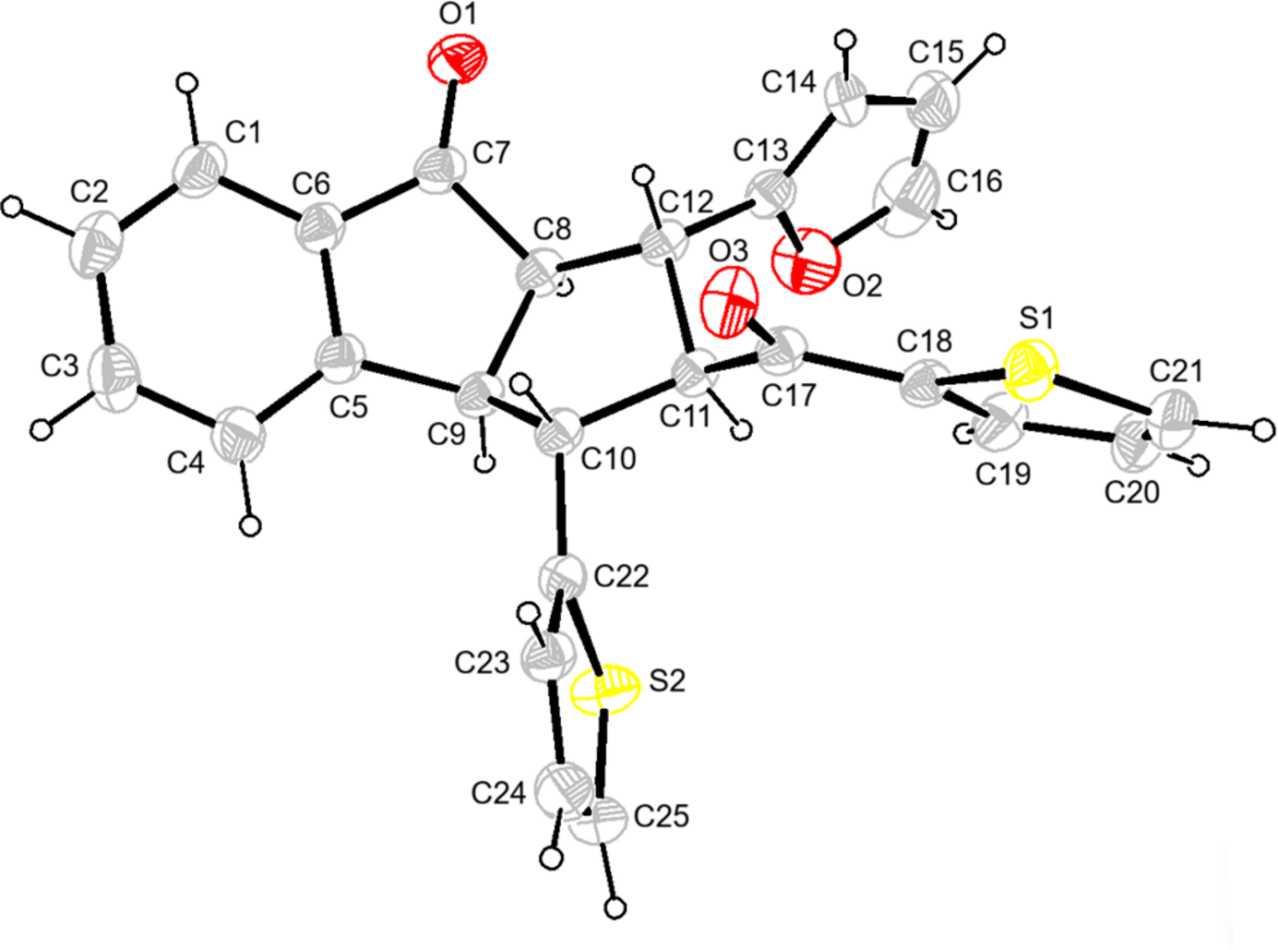
The mol­ecular structure showing the atom labelling scheme and 25% probability level ellipsoids (only the major disorder components being shown).

As illustrated in Fig. 1 ▸, the cyclo­pentane ring (C8–C12) can be described as a five-membered ring twisted on C10—C11 with a conformation very similar to an envelope with puckering parameters (Cremer & Pople, 1975 ▸) *Q*(2) = 0.374 (3) Å and φ(2) = 278.3 (5)°. In the twelve-membered fused triple ring system (C1–C12), the root-mean-square plane (r.m.s. deviation of fitted atoms = 0.0211 Å) of the first two quite planar rings (C1—C9) forms angles of 55.0 (2), 85.4 (1), and 58.8 (1)° with the furan ring (O2/C13–C16) and two thio­phene rings (S1/C18–C21 and S2/C22–C25), respectively. The dihedral angle between the thio­phene rings (S1/C18–C21 and S2/C22–C25) is 72.9 (1)°, while the furan ring (O2/C13–C16) forms angles of 55.7 (2) and 54.0 (2)° with the thio­phene rings S1/C18–C21 and S2/C22–C25, respectively. All geometric parameters are normal and consistent with those of related compounds listed in the *Database survey* section. Each molecule contains five stereogenic (chiral) centres: in the asymmetric unit, C8, C9, C10, C11 and C12 have *R*, *S*, *R*, *R* and *S* configurations, respectively.

## 3. Supra­molecular features and Hirshfeld surface analysis

**Figure 2:**
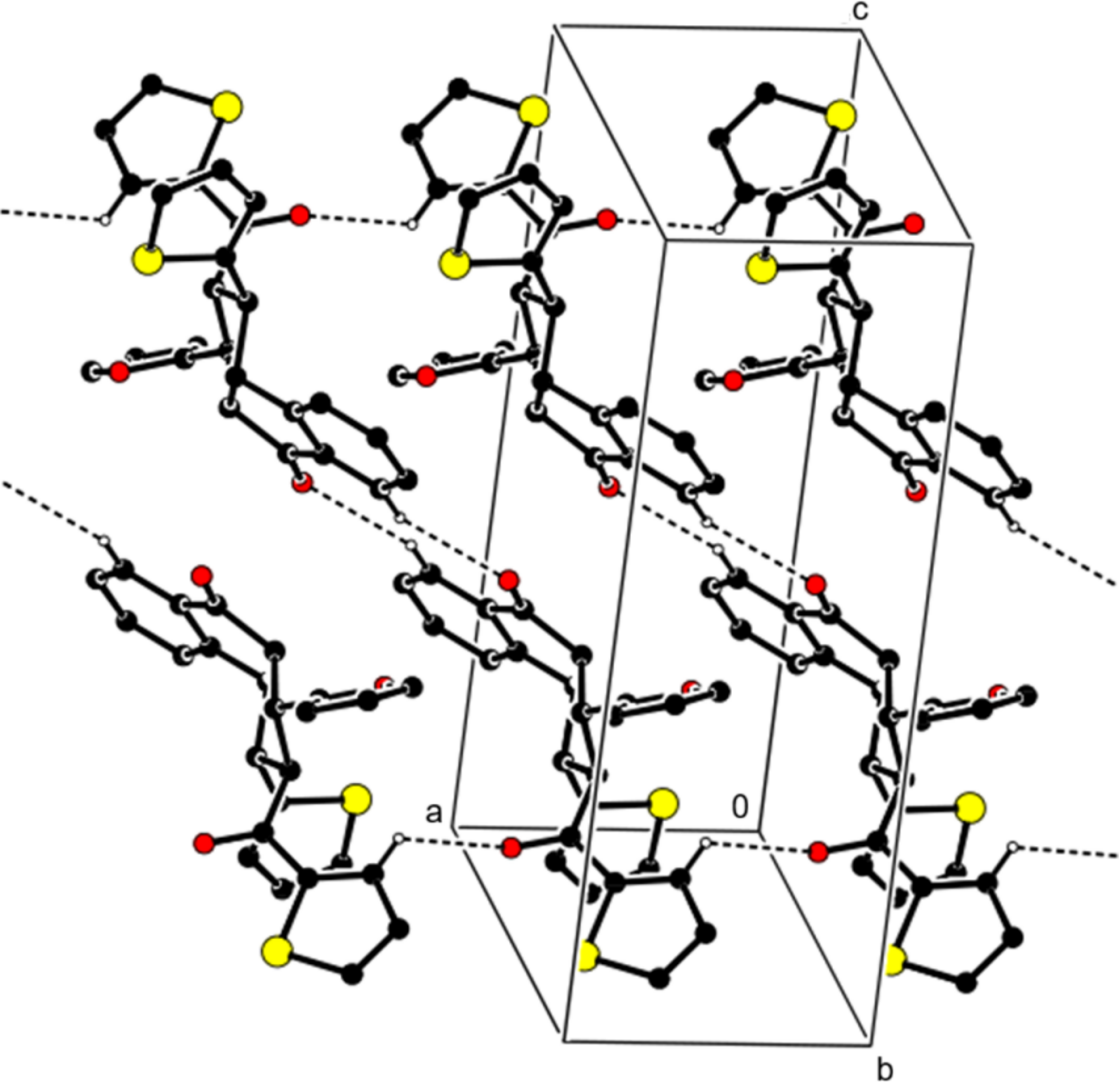
Crystal packing showing the C—H⋯O inter­actions. H atoms not involved in these inter­actions and the minor disorder components have been omitted for clarity.

**Figure 3:**
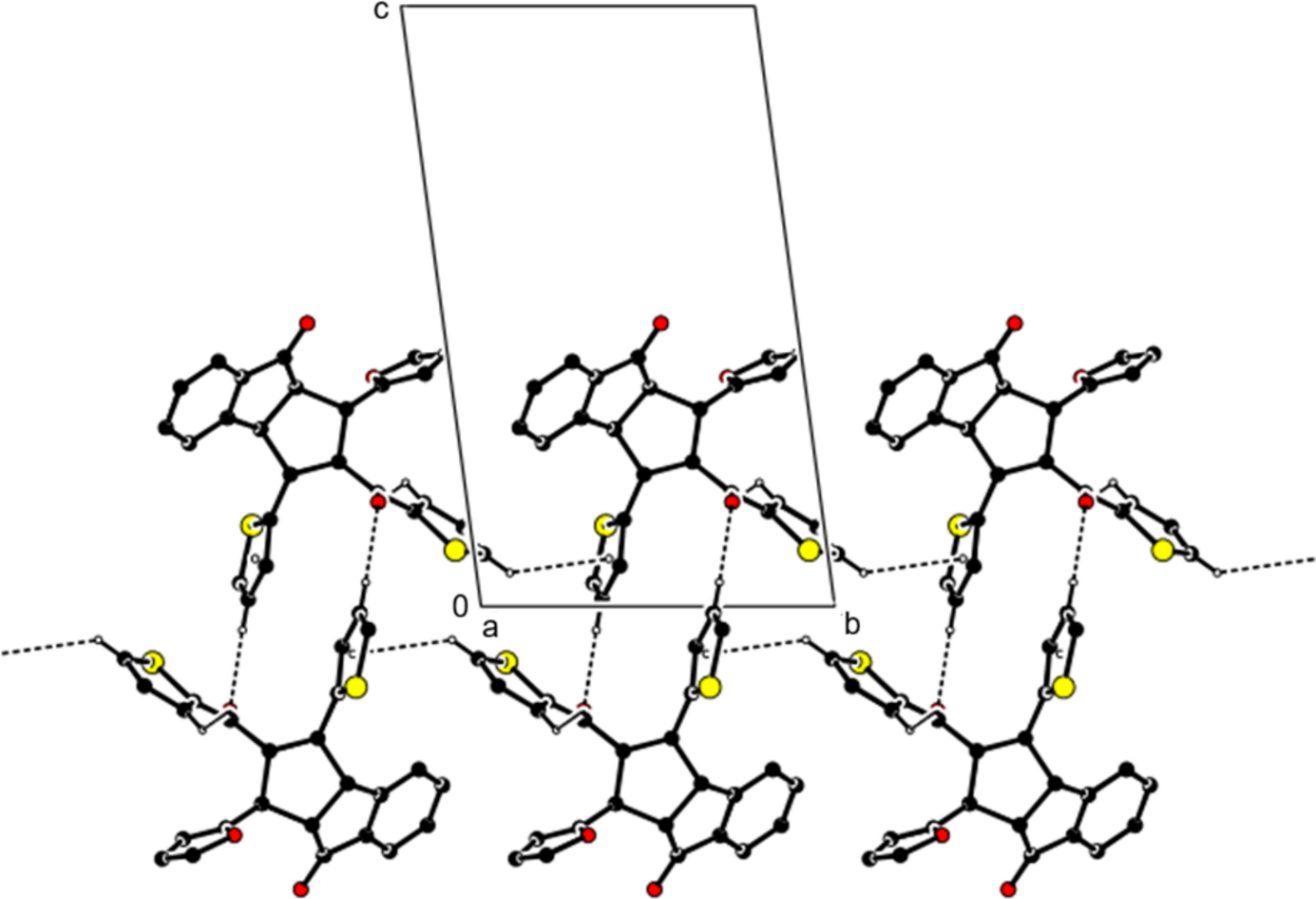
Crystal packing along the *a* axis showing the C—H⋯O and C—H⋯π inter­actions. H atoms not involved in these inter­actions and the minor disorder components have been omitted for clarity.

In the crystal, pairs of enanti­omers are linked by C—H⋯O inter­actions, forming 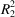
(10) ring motifs (Bernstein *et al.*, 1995 ▸, Fig. 2 ▸, Table 1 ▸). Pairs of mol­ecules are also connected by additional C—H⋯O inter­actions, forming ribbons propagating along th*e a*-axis direction (Fig. 2 ▸). In addition, C—H⋯π inter­actions between these ribbons form layers parallel to the (001) plane (Fig. 3 ▸). van der Waals inter­actions between the layers maintain the cohesion of the crystal structure.

**Figure 4:**
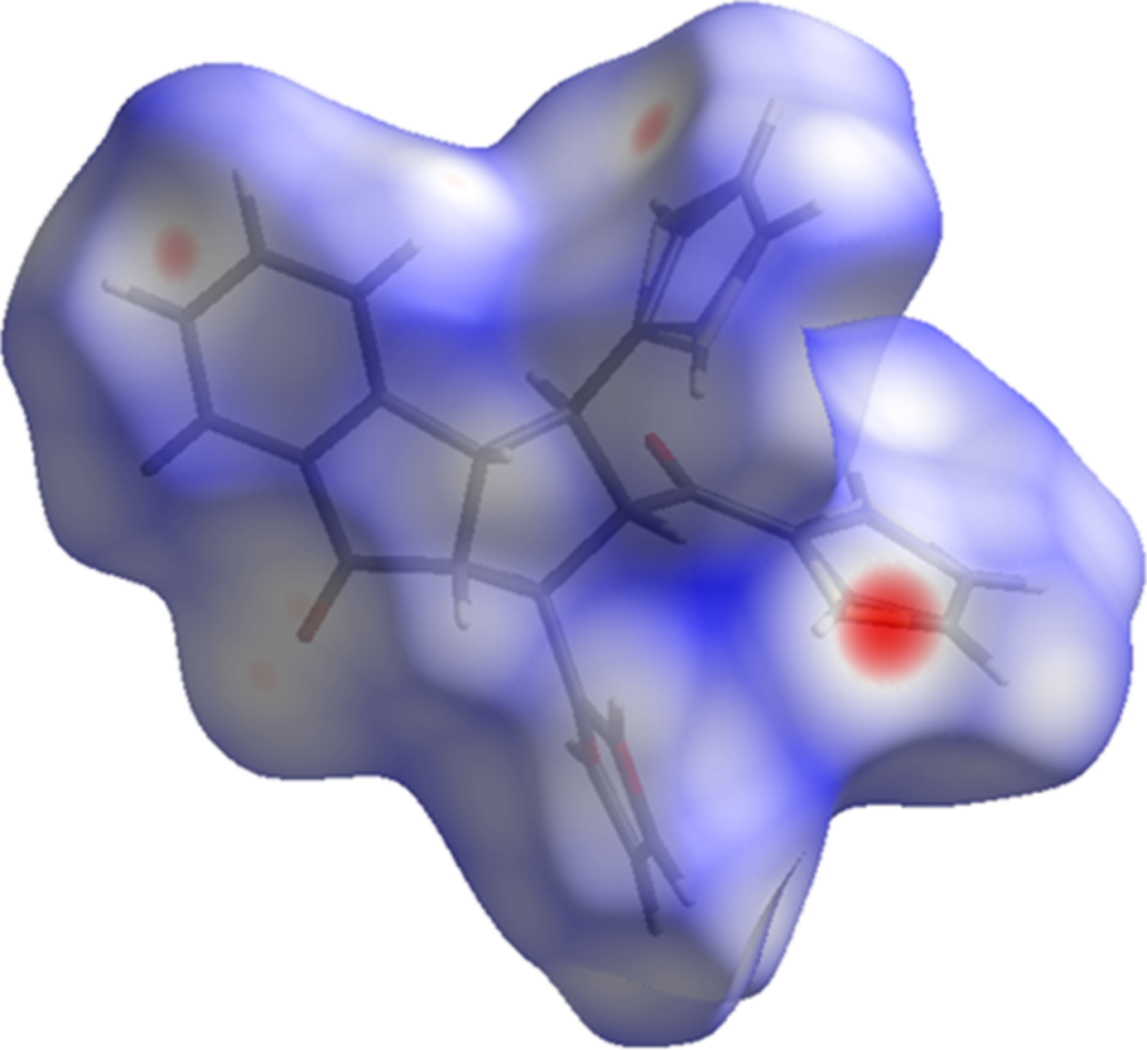
The Hirshfeld surface mapped over *d*_norm_ using a standard surface resolution with a fixed colour scale of −0.2490 (red) to 1.4290 (blue) a.u.

Hirshfeld surfaces and fingerprint plots were generated using *CrystalExplorer* (McKinnon *et al.*, 2007 ▸) to qu­antify and visualize the inter­molecular inter­actions and to explain the observed crystal packing. Hirshfeld surfaces enable the visualization of inter­molecular inter­actions by different colours and colour intensity, representing short or long contacts and indicating the relative strength of the inter­actions. The function *d*_norm_ is a ratio enclosing the distances of any surface point to the nearest inter­ior (*d*_i_) and exterior (*d*_e_) atom and the van der Waals radii of the atoms (Hirshfeld, 1977 ▸; Soman *et al.*, 2014 ▸). The function *d*_norm_ will be equal to zero when inter­molecular distances are close to van der Waals contacts. They are indicated by a white colour on the Hirshfeld surface, while contacts longer than the sum of van der Waals radii with positive *d*_norm_ values are coloured in blue. The surface plot for *d*_norm_ (Fig. 4 ▸) was generated using a high standard surface resolution over a colour scale of −0.25 to 1.43 a.u.

**Figure 5:**
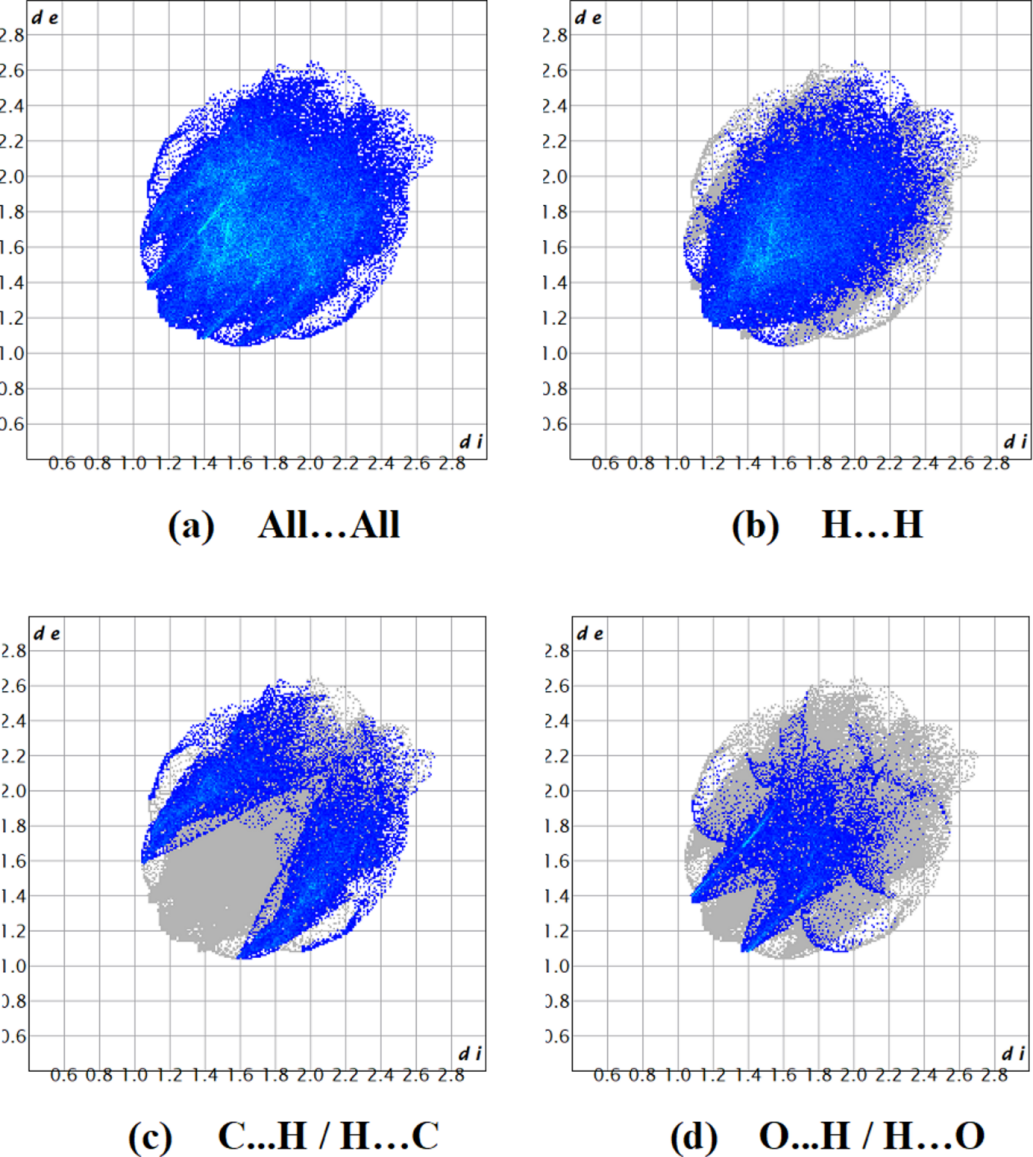
The Hirshfeld surfaces and their associated fingerprint plots, showing (*a*) all inter­actions, and delineated into (*b*) H⋯H, (*c*) C⋯H / H⋯C and (*d*) O⋯H / H⋯O inter­actions [*d*_e_ and *d*_i_ represent the distances from a point on the Hirshfeld surface to the nearest atoms outside (external) and inside (inter­nal) the surface, respectively].

The dark-red spots on the *d*_norm_ surface arise as a result of short inter­atomic contacts (Table 2 ▸), while the other weaker inter­molecular inter­actions appear as light-red spots. The most significant inter­action is H⋯H, contributing 52.8% to the total crystal packing, which is depicted in Fig. 5 ▸*b* as widely distributed points of high density due to the considerable hydrogen content of the mol­ecule with the tip at *d*_e_ = *d*_i_ = 1.15 Å. In the presence of C—H⋯π inter­actions, the pair of typical wings are evident in the fingerprint plot (Fig. 5 ▸*c*) delimited into C⋯·H/H⋯C contacts (26.1%, Table 2 ▸), with the tips at *d*_e_ + *d*_i_ = 2.65 Å. In the fingerprint plot, the O⋯H/H⋯O contacts (Fig. 5 ▸*d*) contribute 14.8% to the Hirshfeld surface and have a distribution of points with tips at *d*_e_ + *d*_i_ = 2.40 Å. Furthermore, there are S⋯H/H⋯S (3.2%), O⋯C/C⋯O (2.0%) and C⋯C (1.1%) contacts.

## 4. Database survey

A search of the Cambridge Structural Database (CSD, Version 6.00, update of April 2025; Groom *et al.*, 2016 ▸) for the 2,3,3a,8a-tetra­hydro­cyclo­penta­[*a*]inden-8(1*H*)-one ring system found one similar compound [dimethyl 1,1-diacetyl-8*a*-hy­droxy-8-oxo-l,2,8,8a-tetra­hydro­cyclo­penta[*a*]indene-2,3-di­carboxyl­ate [(**I**): BIDTIS; Ramazani, 2004 ▸] and two closely related compound, (3,3-dimethyl-1,2,3,4-tetra­hydro­cyclo­penta­[*b*]indole-1,2-dione [(**II**): GAQBAD; Jordon *et al.*, 2012 ▸] and (1*R*,2*S*)-methyl 1-(4-chloro­phen­yl)-3-oxo-1,2,3,4-tetra­hydro­cyclo­penta­[*b*]indole-2-carboxyl­ate [(**IIII**): YAJJEA; Raja & Bolte, 2011 ▸)].

In the crystal of (**I**), only C—H⋯O hydrogen bonds are observed. No π–π stacking inter­actions are observed. In (**II**), the crystal packing is consolidated by N—H⋯O hydrogen bonds, which link the mol­ecules into chains along [10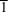], and weak C—H⋯O inter­actions. In (**III**), four of the five mol­ecules form hydrogen-bonded dimers *via* N—H⋯O hydrogen bonds towards another symmetry-independent mol­ecule, whereas the fifth mol­ecule forms a hydrogen-bonded dimer with its symmetry equivalent, also *via* N—H⋯O hydrogen bonds.

## 5. Synthesis and crystallization

The title compound was synthesized according to the reported method (Gezegen *et al.*, 2021 ▸). Crystals were obtained by slow precipitation in an ethanol–di­ethyl ­ether (4:1) solvent mixture.

## 6. Refinement

Crystal data, data collection and structure refinement details are summarized in Table 3 ▸. The aromatic and methyl­ene H atoms were placed at calculated positions with C—H = 0.93 Å and 0.98 Å, respectively and allowed to ride with *U*_iso_(H) = 1.2 *U*_eq_(C). The furan ring (O2/C1–C16) and the two thio­phene rings (S1/C18–C21 and S2/C22–C25) are disordered with ratios of 0.728 (15):0.272 (15), 0.972 (3):0.028 (3) and 0.791 (3):0.209 (3) respectively, around the C—C bond attached to the ring and the terminal cyclo­pentane unit, with a rotation of approximately 180° around the ring. As a result ofthe disorder, the geometries of disordered furan and thio­phene rings were restrained by FLAT and DFIX instructions. The thermal parameters of the atoms of the major components of the disordered furan and thio­phene rings were equaled to those of the corresponding atoms of the minor components using EADP instructions. Twenty one outliers (−2 12 0, −1 12 3, −1 11 6, 0 11 6, −1 0 3, 1 2 1, 0 − 4 3, 2 0 3, −1 − 4 1, −5 − 8 1, −3 − 2 11, 0 9 7, −5 − 8 2, 4 4 3, 0 − 1 21, −1 5 1, 4 − 3 11, −1 1 21, 0 − 8 7, −2 5 14 and 1 − 9 5) were omitted in the last cycles of refinement.

## Supplementary Material

Crystal structure: contains datablock(s) I, global. DOI: 10.1107/S2056989026005141/tx2111sup1.cif

Structure factors: contains datablock(s) I. DOI: 10.1107/S2056989026005141/tx2111Isup2.hkl

Supporting information file. DOI: 10.1107/S2056989026005141/tx2111Isup3.cml

CCDC reference: 2554568

Additional supporting information:  crystallographic information; 3D view; checkCIF report

## Figures and Tables

**Table 1:** Hydrogen-bond geometry (Å, °) *Cg*2 and *Cg*4 are the centroids of the major (S2/C22–C25) and minor (S2*A*/C22*A*–C25*A*) components of the same disordered thio­phene ring.

*D*—H⋯*A*	*D*—H	H⋯*A*	*D*⋯*A*	*D*—H⋯*A*
C24—H24⋯O3^i^	0.93	2.63	3.548 (5)	168
C19—H19⋯O3^ii^	0.93	2.53	3.154 (6)	124
C21—H21⋯*Cg*2^iii^	0.93	2.98	3.710 (4)	137
C21—H21⋯*Cg*4^iii^	0.93	2.95	3.689 (6)	138

Symmetry codes: (i) 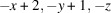; (ii) 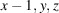; (iii) 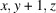.

**Table 2:** Short inter­atomic contacts (Å).

Contact	Distance	Symmetry operation
*H14⋯C3	2.75	*x*, 1 + *y*, *z*
O3⋯*H19	2.53	1 + *x*, *y*, *z*
*S1⋯*S1	3.57	2 − *x*, 2 − *y*, −*z*
H25⋯*C19*A*	2.98	1 − *x*, 1 − *y*, −*z*
*H23⋯*H23	2.52	2 − *x*, 1 − *y*, −*z*
H1⋯O1	2.60	3 − *x*, 1 − *y*, 1 − *z*
C7⋯O1	3.17	2 − *x*, 1 − *y*, 1 − *z*
C15⋯*C14	3.54	2 − *x*, 2 − *y*, 1 − *z*
C15⋯H2	3.04	−1 + *x*, 1 + *y*, *z*
H16⋯C16	3.09	1 − *x*, 2 − *y*, 1 − *z*

The prefix * represent the atom of the minor disordered component.

**Table 3:** Experimental details

Crystal data	
Chemical formula	C_25_H_18_O_3_S_2_
*M*_r_	430.51
Crystal system, space group	Triclinic, *P* 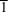
Temperature (K)	293
*a*, *b*, *c* (Å)	6.2444 (4), 10.1570 (6), 17.1745 (11)
α, β, γ (°)	96.604 (5), 94.982 (5), 99.812 (5)
*V* (Å^3^)	1059.90 (12)
*Z*	2
Radiation type	Cu *K*α
μ (mm^−1^)	2.47
Crystal size (mm)	0.15 × 0.11 × 0.09

Data collection	
Diffractometer	Xcalibur, Ruby, Gemini
Absorption correction	Analytical (SCALE3 ABSPACK in *CrysAlis PRO*; Agilent, 2014 ▸; Bourhis *et al.*, 2015 ▸)
*T*_min_, *T*_max_	0.879, 0.912
No. of measured, independent and observed [*I* > 2σ(*I*)] reflections	16801, 4326, 2554
*R*_int_	0.052
(sin θ/λ)_max_ (Å^−1^)	0.629

Refinement	
*R*[*F*^2^ > 2σ(*F*^2^)], *wR*(*F*^2^), *S*	0.053, 0.141, 1.02
No. of reflections	4326
No. of parameters	292
No. of restraints	24
H-atom treatment	H-atom parameters constrained
Δρ_max_, Δρ_min_ (e Å^−3^)	0.18, −0.28

Computer programs: *CrysAlis PRO* (Agilent, 2014 ▸), *SHELXS97* (Sheldrick, 2008 ▸), *SHELXL2014* (Sheldrick, 2015 ▸), *ORTEP-3 for Windows* (Farrugia, 2012 ▸) and *PLATON* (Spek, 2020 ▸).

## supplementary crystallographic information

### 1-(Furan-2-yl)-2-(thiophene-2-carbonyl)-3-(thiophen-2-yl)-2,3,3a,8a-tetrahydrocyclopenta[a]inden-8(1H)-one Crystal data

**Table:**

C_25_H_18_O_3_S_2_	*Z* = 2
*M**_r_* = 430.51	*F*(000) = 448
Triclinic, *P*1	*D*_x_ = 1.349 Mg m^−^^3^
*a* = 6.2444 (4) Å	Cu *K*α radiation, λ = 1.54184 Å
*b* = 10.1570 (6) Å	Cell parameters from 2960 reflections
*c* = 17.1745 (11) Å	θ = 2.6–60.2°
α = 96.604 (5)°	µ = 2.47 mm^−^^1^
β = 94.982 (5)°	*T* = 293 K
γ = 99.812 (5)°	Prism, brown
*V* = 1059.90 (12) Å^3^	0.15 × 0.11 × 0.09 mm

### 1-(Furan-2-yl)-2-(thiophene-2-carbonyl)-3-(thiophen-2-yl)-2,3,3a,8a-tetrahydrocyclopenta[a]inden-8(1H)-one Data collection

**Table:**

Xcalibur, Ruby, Gemini diffractometer	4326 independent reflections
Radiation source: fine-focus sealed X-ray tube, Enhance (Cu) X-ray Source	2554 reflections with *I* > 2σ(*I*)
Graphite monochromator	*R*_int_ = 0.052
Detector resolution: 10.2673 pixels mm^-1^	θ_max_ = 76.0°, θ_min_ = 2.6°
ω scans	*h* = −7→7
Absorption correction: analytical (SCALE3 ABSPACK in CrysAlisPro; Agilent, 2014; Bourhis *et al.*, 2015)	*k* = −11→12
*T*_min_ = 0.879, *T*_max_ = 0.912	*l* = −21→21
16801 measured reflections	

### 1-(Furan-2-yl)-2-(thiophene-2-carbonyl)-3-(thiophen-2-yl)-2,3,3a,8a-tetrahydrocyclopenta[a]inden-8(1H)-one Refinement

**Table:**

Refinement on *F*^2^	24 restraints
Least-squares matrix: full	Hydrogen site location: inferred from neighbouring sites
*R*[*F*^2^ > 2σ(*F*^2^)] = 0.053	H-atom parameters constrained
*wR*(*F*^2^) = 0.141	*w* = 1/[σ^2^(*F*_o_^2^) + (0.0497*P*)^2^ + 0.3219*P*] where *P* = (*F*_o_^2^ + 2*F*_c_^2^)/3
*S* = 1.02	(Δ/σ)_max_ < 0.001
4326 reflections	Δρ_max_ = 0.18 e Å^−^^3^
292 parameters	Δρ_min_ = −0.28 e Å^−^^3^

### 1-(Furan-2-yl)-2-(thiophene-2-carbonyl)-3-(thiophen-2-yl)-2,3,3a,8a-tetrahydrocyclopenta[a]inden-8(1H)-one Special details

**Table:**

Geometry. All esds (except the esd in the dihedral angle between two l.s. planes) are estimated using the full covariance matrix. The cell esds are taken into account individually in the estimation of esds in distances, angles and torsion angles; correlations between esds in cell parameters are only used when they are defined by crystal symmetry. An approximate (isotropic) treatment of cell esds is used for estimating esds involving l.s. planes.

### 1-(Furan-2-yl)-2-(thiophene-2-carbonyl)-3-(thiophen-2-yl)-2,3,3a,8a-tetrahydrocyclopenta[a]inden-8(1H)-one Fractional atomic coordinates and isotropic or equivalent isotropic displacement parameters (Å^2^)

**Table:**

	*x*	*y*	*z*	*U*_iso_*/*U*_eq_	Occ. (<1)
C1	1.3333 (6)	0.3504 (4)	0.4078 (2)	0.0816 (10)	
H1	1.425631	0.387974	0.453205	0.098*	
C2	1.3627 (6)	0.2335 (4)	0.3648 (3)	0.0912 (12)	
H2	1.477643	0.192023	0.380908	0.109*	
C3	1.2234 (7)	0.1767 (4)	0.2979 (2)	0.0943 (12)	
H3	1.245240	0.096998	0.269859	0.113*	
C4	1.0519 (6)	0.2368 (4)	0.2719 (2)	0.0821 (10)	
H4	0.958834	0.198227	0.226784	0.099*	
C5	1.0221 (5)	0.3548 (3)	0.31416 (17)	0.0622 (8)	
C6	1.1617 (5)	0.4111 (3)	0.38168 (18)	0.0639 (8)	
C7	1.1025 (5)	0.5376 (3)	0.41473 (18)	0.0664 (8)	
C8	0.9029 (5)	0.5597 (3)	0.36442 (16)	0.0585 (7)	
H8	0.777415	0.555134	0.395187	0.070*	
C9	0.8586 (4)	0.4436 (3)	0.29527 (16)	0.0566 (7)	
H9	0.708672	0.393492	0.291977	0.068*	
C10	0.8963 (4)	0.5109 (3)	0.21971 (15)	0.0548 (7)	
H10	1.052443	0.520864	0.213287	0.066*	
C11	0.8460 (4)	0.6523 (3)	0.24090 (15)	0.0530 (7)	
H11	0.687391	0.648311	0.235579	0.064*	
C12	0.9393 (4)	0.6935 (3)	0.32874 (15)	0.0548 (7)	
H12	1.097356	0.724235	0.330431	0.066*	
C13	0.8475 (5)	0.8043 (4)	0.36928 (17)	0.0637 (8)	
C15	0.7761 (11)	0.9961 (5)	0.4206 (3)	0.136 (2)	
H15	0.794904	1.086055	0.442280	0.163*	
C16	0.5983 (11)	0.9082 (7)	0.4144 (3)	0.138 (2)	
H16	0.466058	0.926111	0.429993	0.166*	
C17	0.9511 (4)	0.7510 (3)	0.18900 (16)	0.0564 (7)	
C18	0.8314 (4)	0.8479 (3)	0.15847 (15)	0.0551 (7)	
C20	0.5753 (6)	0.9811 (4)	0.1325 (2)	0.0852 (11)	
H20	0.445928	1.013820	0.136708	0.102*	
C21	0.7285 (6)	1.0266 (4)	0.0874 (2)	0.0823 (10)	
H21	0.716259	1.094166	0.055939	0.099*	
C22	0.7719 (5)	0.4364 (3)	0.14439 (17)	0.0611 (8)	
C24	0.6832 (8)	0.3449 (4)	0.0115 (2)	0.0927 (12)	
H24	0.707883	0.316855	−0.039875	0.111*	
C25	0.4913 (7)	0.3273 (4)	0.0388 (2)	0.0959 (13)	
H25	0.362730	0.287032	0.007420	0.115*	
O2	0.6319 (8)	0.7833 (6)	0.3813 (4)	0.1119 (18)	0.728 (15)
C14	0.9410 (13)	0.9294 (6)	0.3876 (6)	0.096 (3)	0.728 (15)
H14	1.083325	0.967919	0.380998	0.115*	0.728 (15)
O2A	0.9442 (17)	0.9357 (11)	0.3889 (12)	0.1119 (18)	0.272 (15)
C14A	0.640 (3)	0.7812 (11)	0.3806 (14)	0.096 (3)	0.272 (15)
H14A	0.542360	0.699876	0.369116	0.115*	0.272 (15)
S1	0.94622 (14)	0.94899 (10)	0.09363 (5)	0.0705 (3)	0.972 (3)
C19	0.6344 (10)	0.8776 (6)	0.1725 (3)	0.0759 (15)	0.972 (3)
H19	0.545669	0.833432	0.205825	0.091*	0.972 (3)
S1A	0.598 (13)	0.867 (4)	0.1851 (15)	0.0705 (3)	0.028 (3)
C19A	0.907 (3)	0.940 (7)	0.103 (4)	0.0759 (15)	0.028 (3)
H19A	1.038509	0.945780	0.080668	0.091*	0.028 (3)
S2	0.4979 (2)	0.3835 (2)	0.13452 (8)	0.0902 (5)	0.791 (3)
C23	0.8340 (9)	0.4075 (7)	0.0673 (4)	0.0619 (13)	0.791 (3)
H23	0.977914	0.432537	0.056898	0.074*	0.791 (3)
S2A	0.9015 (9)	0.4127 (12)	0.0703 (6)	0.0902 (5)	0.209 (3)
C23A	0.5356 (15)	0.383 (2)	0.1228 (7)	0.0619 (13)	0.209 (3)
H23A	0.429679	0.383924	0.157582	0.074*	0.209 (3)
O1	1.1966 (4)	0.6156 (3)	0.47151 (13)	0.0895 (7)	
O3	1.1381 (3)	0.7493 (2)	0.17392 (14)	0.0796 (7)	

### 1-(Furan-2-yl)-2-(thiophene-2-carbonyl)-3-(thiophen-2-yl)-2,3,3a,8a-tetrahydrocyclopenta[a]inden-8(1H)-one Atomic displacement parameters (Å^2^)

**Table:**

	*U*^11^	*U*^22^	*U*^33^	*U*^12^	*U*^13^	*U*^23^
C1	0.076 (2)	0.085 (3)	0.085 (2)	0.014 (2)	−0.0071 (18)	0.031 (2)
C2	0.082 (2)	0.094 (3)	0.108 (3)	0.027 (2)	0.009 (2)	0.044 (3)
C3	0.124 (3)	0.075 (3)	0.096 (3)	0.038 (2)	0.018 (3)	0.027 (2)
C4	0.106 (3)	0.069 (2)	0.072 (2)	0.019 (2)	−0.0013 (19)	0.0149 (19)
C5	0.0681 (18)	0.063 (2)	0.0565 (17)	0.0077 (16)	0.0032 (14)	0.0200 (15)
C6	0.0651 (18)	0.065 (2)	0.0632 (18)	0.0074 (16)	0.0019 (14)	0.0240 (16)
C7	0.0725 (19)	0.073 (2)	0.0522 (17)	0.0060 (17)	−0.0020 (15)	0.0195 (16)
C8	0.0603 (16)	0.068 (2)	0.0488 (15)	0.0102 (15)	0.0084 (13)	0.0167 (14)
C9	0.0551 (15)	0.0635 (19)	0.0511 (15)	0.0058 (14)	0.0026 (12)	0.0170 (14)
C10	0.0530 (15)	0.0620 (19)	0.0506 (15)	0.0093 (14)	0.0056 (12)	0.0146 (14)
C11	0.0491 (14)	0.0603 (18)	0.0512 (15)	0.0090 (13)	0.0067 (12)	0.0151 (13)
C12	0.0511 (14)	0.0660 (19)	0.0479 (15)	0.0083 (14)	0.0061 (12)	0.0129 (14)
C13	0.0654 (18)	0.076 (2)	0.0516 (17)	0.0171 (17)	0.0055 (14)	0.0106 (16)
C15	0.194 (6)	0.095 (4)	0.113 (4)	0.067 (4)	−0.050 (4)	−0.021 (3)
C16	0.160 (5)	0.183 (6)	0.098 (4)	0.106 (5)	0.037 (4)	0.002 (4)
C17	0.0537 (16)	0.0628 (19)	0.0517 (15)	0.0061 (14)	0.0045 (12)	0.0119 (14)
C18	0.0536 (15)	0.0619 (19)	0.0483 (15)	0.0046 (14)	0.0025 (12)	0.0117 (13)
C20	0.070 (2)	0.089 (3)	0.102 (3)	0.0253 (19)	−0.0017 (19)	0.027 (2)
C21	0.090 (2)	0.071 (2)	0.086 (2)	0.013 (2)	−0.009 (2)	0.029 (2)
C22	0.0719 (18)	0.0576 (19)	0.0547 (17)	0.0141 (15)	−0.0008 (14)	0.0137 (14)
C24	0.146 (4)	0.075 (3)	0.056 (2)	0.026 (3)	−0.005 (2)	0.0099 (19)
C25	0.107 (3)	0.079 (3)	0.089 (3)	0.015 (2)	−0.035 (2)	−0.006 (2)
O2	0.080 (3)	0.131 (4)	0.129 (4)	0.031 (3)	0.036 (3)	−0.005 (3)
C14	0.109 (6)	0.047 (3)	0.126 (6)	0.010 (3)	−0.028 (5)	0.021 (4)
O2A	0.080 (3)	0.131 (4)	0.129 (4)	0.031 (3)	0.036 (3)	−0.005 (3)
C14A	0.109 (6)	0.047 (3)	0.126 (6)	0.010 (3)	−0.028 (5)	0.021 (4)
S1	0.0778 (6)	0.0703 (6)	0.0667 (5)	0.0094 (4)	0.0098 (4)	0.0271 (4)
C19	0.064 (3)	0.090 (3)	0.077 (3)	0.0114 (19)	0.009 (2)	0.029 (2)
S1A	0.0778 (6)	0.0703 (6)	0.0667 (5)	0.0094 (4)	0.0098 (4)	0.0271 (4)
C19A	0.064 (3)	0.090 (3)	0.077 (3)	0.0114 (19)	0.009 (2)	0.029 (2)
S2	0.0680 (7)	0.1151 (11)	0.0765 (9)	0.0043 (7)	−0.0041 (6)	−0.0058 (8)
C23	0.063 (3)	0.064 (3)	0.057 (3)	0.005 (3)	0.003 (3)	0.016 (2)
S2A	0.0680 (7)	0.1151 (11)	0.0765 (9)	0.0043 (7)	−0.0041 (6)	−0.0058 (8)
C23A	0.063 (3)	0.064 (3)	0.057 (3)	0.005 (3)	0.003 (3)	0.016 (2)
O1	0.1049 (18)	0.0877 (18)	0.0680 (15)	0.0115 (14)	−0.0216 (13)	0.0098 (13)
O3	0.0608 (13)	0.0917 (17)	0.0993 (17)	0.0199 (12)	0.0297 (12)	0.0424 (14)

### 1-(Furan-2-yl)-2-(thiophene-2-carbonyl)-3-(thiophen-2-yl)-2,3,3a,8a-tetrahydrocyclopenta[a]inden-8(1H)-one Geometric parameters (Å, º)

**Table:**

C1—C2	1.372 (5)	C15—H15	0.9300
C1—C6	1.391 (4)	C16—O2	1.387 (8)
C1—H1	0.9300	C16—C14A	1.429 (10)
C2—C3	1.384 (5)	C16—H16	0.9300
C2—H2	0.9300	C17—O3	1.220 (3)
C3—C4	1.386 (5)	C17—C18	1.450 (4)
C3—H3	0.9300	C18—C19	1.351 (7)
C4—C5	1.378 (5)	C18—C19A	1.46 (9)
C4—H4	0.9300	C18—S1A	1.60 (9)
C5—C6	1.390 (4)	C18—S1	1.720 (3)
C5—C9	1.511 (4)	C20—C21	1.337 (5)
C6—C7	1.463 (5)	C20—C19	1.402 (7)
C7—O1	1.220 (4)	C20—S1A	1.56 (4)
C7—C8	1.518 (4)	C20—H20	0.9300
C8—C12	1.545 (4)	C21—C19A	1.56 (5)
C8—C9	1.545 (4)	C21—S1	1.685 (4)
C8—H8	0.9800	C21—H21	0.9300
C9—C10	1.552 (4)	C22—C23	1.424 (7)
C9—H9	0.9800	C22—C23A	1.482 (8)
C10—C22	1.500 (4)	C22—S2A	1.583 (10)
C10—C11	1.533 (4)	C22—S2	1.691 (3)
C10—H10	0.9800	C24—C23	1.307 (7)
C11—C17	1.521 (4)	C24—C25	1.315 (5)
C11—C12	1.552 (4)	C24—S2A	1.623 (8)
C11—H11	0.9800	C24—H24	0.9300
C12—C13	1.480 (4)	C25—C23A	1.471 (9)
C12—H12	0.9800	C25—S2	1.672 (4)
C13—C14	1.295 (7)	C25—H25	0.9300
C13—C14A	1.310 (15)	C14—H14	0.9300
C13—O2A	1.358 (9)	C14A—H14A	0.9300
C13—O2	1.364 (6)	C19—H19	0.9300
C15—C16	1.288 (7)	C19A—H19A	0.9300
C15—O2A	1.420 (8)	C23—H23	0.9300
C15—C14	1.446 (10)	C23A—H23A	0.9300
			
C2—C1—C6	118.3 (3)	C14—C15—H15	126.2
C2—C1—H1	120.9	C15—C16—O2	110.5 (5)
C6—C1—H1	120.9	C15—C16—C14A	108.6 (7)
C1—C2—C3	120.8 (4)	C15—C16—H16	124.8
C1—C2—H2	119.6	O2—C16—H16	124.8
C3—C2—H2	119.6	O3—C17—C18	120.1 (3)
C2—C3—C4	121.0 (4)	O3—C17—C11	119.6 (3)
C2—C3—H3	119.5	C18—C17—C11	120.3 (2)
C4—C3—H3	119.5	C19—C18—C17	131.0 (3)
C5—C4—C3	118.6 (4)	C17—C18—C19A	125.4 (12)
C5—C4—H4	120.7	C17—C18—S1A	122.1 (14)
C3—C4—H4	120.7	C19A—C18—S1A	112.4 (9)
C4—C5—C6	120.2 (3)	C19—C18—S1	109.8 (3)
C4—C5—C9	128.7 (3)	C17—C18—S1	119.2 (2)
C6—C5—C9	111.1 (3)	C21—C20—C19	111.4 (4)
C5—C6—C1	121.1 (3)	C21—C20—S1A	123 (3)
C5—C6—C7	110.2 (3)	C21—C20—H20	124.3
C1—C6—C7	128.7 (3)	C19—C20—H20	124.3
O1—C7—C6	127.3 (3)	C20—C21—C19A	103 (3)
O1—C7—C8	124.5 (3)	C20—C21—S1	113.0 (3)
C6—C7—C8	108.2 (3)	C20—C21—H21	123.5
C7—C8—C12	113.5 (2)	S1—C21—H21	123.5
C7—C8—C9	105.6 (3)	C23—C22—C10	132.4 (3)
C12—C8—C9	107.5 (2)	C23A—C22—C10	131.5 (5)
C7—C8—H8	110.1	C23A—C22—S2A	110.0 (5)
C12—C8—H8	110.1	C10—C22—S2A	118.4 (3)
C9—C8—H8	110.1	C23—C22—S2	104.6 (3)
C5—C9—C8	104.7 (2)	C10—C22—S2	122.6 (2)
C5—C9—C10	113.4 (2)	C23—C24—C25	110.0 (4)
C8—C9—C10	106.0 (2)	C25—C24—S2A	120.0 (5)
C5—C9—H9	110.8	C23—C24—H24	125.0
C8—C9—H9	110.8	C25—C24—H24	125.0
C10—C9—H9	110.8	C24—C25—C23A	105.1 (4)
C22—C10—C11	113.8 (2)	C24—C25—S2	114.4 (3)
C22—C10—C9	116.2 (2)	C24—C25—H25	122.8
C11—C10—C9	103.6 (2)	S2—C25—H25	122.8
C22—C10—H10	107.6	C13—O2—C16	104.2 (5)
C11—C10—H10	107.6	C13—C14—C15	105.0 (6)
C9—C10—H10	107.6	C13—C14—H14	127.5
C17—C11—C10	111.7 (2)	C15—C14—H14	127.5
C17—C11—C12	111.4 (2)	C13—O2A—C15	103.1 (6)
C10—C11—C12	104.7 (2)	C13—C14A—C16	104.8 (7)
C17—C11—H11	109.6	C13—C14A—H14A	127.6
C10—C11—H11	109.6	C16—C14A—H14A	127.6
C12—C11—H11	109.6	C21—S1—C18	91.65 (16)
C13—C12—C8	115.6 (2)	C18—C19—C20	114.1 (4)
C13—C12—C11	113.8 (2)	C18—C19—H19	123.0
C8—C12—C11	104.0 (2)	C20—C19—H19	123.0
C13—C12—H12	107.7	C20—S1A—C18	93.7 (18)
C8—C12—H12	107.7	C18—C19A—C21	107.9 (17)
C11—C12—H12	107.7	C18—C19A—H19A	126.1
C14A—C13—O2A	113.3 (6)	C21—C19A—H19A	126.1
C14—C13—O2	112.5 (5)	C25—S2—C22	92.6 (2)
C14—C13—C12	127.4 (5)	C24—C23—C22	118.2 (5)
C14A—C13—C12	118.3 (6)	C24—C23—H23	120.9
O2A—C13—C12	127.9 (4)	C22—C23—H23	120.9
O2—C13—C12	119.5 (4)	C22—S2A—C24	94.0 (4)
C16—C15—O2A	109.9 (6)	C25—C23A—C22	110.8 (6)
C16—C15—C14	107.5 (5)	C25—C23A—H23A	124.6
C16—C15—H15	126.2	C22—C23A—H23A	124.6
			
C6—C1—C2—C3	−0.9 (5)	O3—C17—C18—C19	−173.1 (4)
C1—C2—C3—C4	0.7 (6)	C11—C17—C18—C19	6.0 (5)
C2—C3—C4—C5	−0.1 (6)	O3—C17—C18—C19A	6.3 (17)
C3—C4—C5—C6	−0.3 (5)	C11—C17—C18—C19A	−174.6 (17)
C3—C4—C5—C9	175.6 (3)	O3—C17—C18—S1A	−172.1 (14)
C4—C5—C6—C1	0.1 (5)	C11—C17—C18—S1A	7.0 (14)
C9—C5—C6—C1	−176.5 (3)	O3—C17—C18—S1	6.0 (4)
C4—C5—C6—C7	178.1 (3)	C11—C17—C18—S1	−174.8 (2)
C9—C5—C6—C7	1.6 (3)	S1A—C20—C21—C19A	0.3 (19)
C2—C1—C6—C5	0.5 (5)	C19—C20—C21—S1	−1.1 (5)
C2—C1—C6—C7	−177.1 (3)	C11—C10—C22—C23	104.1 (5)
C5—C6—C7—O1	−176.4 (3)	C9—C10—C22—C23	−135.7 (4)
C1—C6—C7—O1	1.4 (5)	C11—C10—C22—C23A	−67.1 (13)
C5—C6—C7—C8	1.9 (3)	C9—C10—C22—C23A	53.1 (13)
C1—C6—C7—C8	179.7 (3)	C11—C10—C22—S2A	109.4 (5)
O1—C7—C8—C12	56.5 (4)	C9—C10—C22—S2A	−130.4 (5)
C6—C7—C8—C12	−121.9 (3)	C11—C10—C22—S2	−67.7 (3)
O1—C7—C8—C9	173.9 (3)	C9—C10—C22—S2	52.5 (4)
C6—C7—C8—C9	−4.4 (3)	S2A—C24—C25—C23A	1.3 (11)
C4—C5—C9—C8	179.5 (3)	C23—C24—C25—S2	−2.0 (5)
C6—C5—C9—C8	−4.3 (3)	C14—C13—O2—C16	−3.6 (8)
C4—C5—C9—C10	−65.4 (4)	C12—C13—O2—C16	−175.6 (4)
C6—C5—C9—C10	110.8 (3)	C15—C16—O2—C13	0.8 (8)
C7—C8—C9—C5	5.1 (3)	O2—C13—C14—C15	4.6 (9)
C12—C8—C9—C5	126.5 (2)	C12—C13—C14—C15	175.8 (4)
C7—C8—C9—C10	−115.0 (2)	C16—C15—C14—C13	−3.9 (9)
C12—C8—C9—C10	6.4 (3)	C14A—C13—O2A—C15	4 (2)
C5—C9—C10—C22	92.8 (3)	C12—C13—O2A—C15	175.8 (5)
C8—C9—C10—C22	−152.9 (2)	C16—C15—O2A—C13	−3.8 (15)
C5—C9—C10—C11	−141.6 (2)	O2A—C13—C14A—C16	−3 (2)
C8—C9—C10—C11	−27.3 (3)	C12—C13—C14A—C16	−175.4 (8)
C22—C10—C11—C17	−74.2 (3)	C15—C16—C14A—C13	0.3 (18)
C9—C10—C11—C17	158.7 (2)	C20—C21—S1—C18	0.7 (3)
C22—C10—C11—C12	165.1 (2)	C19—C18—S1—C21	−0.1 (3)
C9—C10—C11—C12	38.1 (3)	C17—C18—S1—C21	−179.4 (2)
C7—C8—C12—C13	−101.4 (3)	C17—C18—C19—C20	178.7 (3)
C9—C8—C12—C13	142.3 (2)	S1—C18—C19—C20	−0.5 (5)
C7—C8—C12—C11	133.1 (3)	C21—C20—C19—C18	1.0 (5)
C9—C8—C12—C11	16.8 (3)	C21—C20—S1A—C18	−1 (2)
C17—C11—C12—C13	78.4 (3)	C17—C18—S1A—C20	179.3 (6)
C10—C11—C12—C13	−160.7 (2)	C19A—C18—S1A—C20	1 (2)
C17—C11—C12—C8	−154.9 (2)	C17—C18—C19A—C21	−179.2 (8)
C10—C11—C12—C8	−34.0 (3)	S1A—C18—C19A—C21	−1 (3)
C8—C12—C13—C14	135.5 (6)	C20—C21—C19A—C18	0 (2)
C11—C12—C13—C14	−104.2 (6)	C24—C25—S2—C22	−0.1 (4)
C8—C12—C13—C14A	−53.4 (13)	C23—C22—S2—C25	1.9 (4)
C11—C12—C13—C14A	67.0 (13)	C10—C22—S2—C25	175.7 (3)
C8—C12—C13—O2A	135.4 (12)	C25—C24—C23—C22	3.8 (7)
C11—C12—C13—O2A	−104.3 (12)	C10—C22—C23—C24	−176.6 (4)
C8—C12—C13—O2	−53.8 (5)	S2—C22—C23—C24	−3.7 (6)
C11—C12—C13—O2	66.5 (5)	C23A—C22—S2A—C24	2.0 (13)
C14—C15—C16—O2	1.9 (9)	C10—C22—S2A—C24	−175.2 (3)
O2A—C15—C16—C14A	2.3 (15)	C25—C24—S2A—C22	−2.2 (8)
C10—C11—C17—O3	−42.4 (4)	C24—C25—C23A—C22	0.3 (17)
C12—C11—C17—O3	74.2 (4)	C10—C22—C23A—C25	175.0 (6)
C10—C11—C17—C18	138.4 (3)	S2A—C22—C23A—C25	−1.7 (19)
C12—C11—C17—C18	−104.9 (3)		

### 1-(Furan-2-yl)-2-(thiophene-2-carbonyl)-3-(thiophen-2-yl)-2,3,3a,8a-tetrahydrocyclopenta[a]inden-8(1H)-one Hydrogen-bond geometry (Å, º)

*Cg*2 and *Cg*4 are the centroids of the major (S2/C22–C25) and minor (S2*A*/C22*A*–C25*A*) components of the same disordered thiophene ring.

**Table:**

*D*—H···*A*	*D*—H	H···*A*	*D*···*A*	*D*—H···*A*
C24—H24···O3^i^	0.93	2.63	3.548 (5)	168
C19—H19···O3^ii^	0.93	2.53	3.154 (6)	124
C21—H21···*Cg*2^iii^	0.93	2.98	3.710 (4)	137
C21—H21···*Cg*4^iii^	0.93	2.95	3.689 (6)	138

Symmetry codes: (i) −*x*+2, −*y*+1, −*z*; (ii) *x*−1, *y*, *z*; (iii) *x*, *y*+1, *z*.
